# Kanamycin inhibits daidzein metabolism and abilities of the metabolites to prevent bone loss in ovariectomized mice

**DOI:** 10.1186/s13104-016-2139-7

**Published:** 2016-07-07

**Authors:** Shin-ichi Katsumata, Maiko Fujioka, Shungo Fujii, Ken Takeda, Yoshiko Ishimi, Mariko Uehara

**Affiliations:** Department of Nutritional Science, Faculty of Applied Bioscience, Tokyo University of Agriculture, 1-1-1 Sakuragaoka, Setagaya-ku, Tokyo, 156-8502 Japan; Department of Food Function and Labeling, National Institute of Health and Nutrition, National Institutes of Biomedical Innovation and Health and Nutrition, 1-23-1 Toyama, Shinjuku-ku, Tokyo, 162-8636 Japan; Department of Hygienic Chemistry, Faculty of Pharmaceutical Sciences, Tokyo University of Science (TUS), 2641 Yamazaki, Noda, Chiba, 278-8510 Japan; Department of Nutritional Science and Food Safety, Faculty of Applied Bioscience, Tokyo University of Agriculture, 1-1-1 Sakuragaoka, Setagaya-ku, Tokyo, 156-8502 Japan

**Keywords:** Daidzein, Equol, *O*-desmethylangolensin, Kanamycin, Bone mineral density, Ovariectomy

## Abstract

**Background:**

Daidzein is an isoflavone derived from soybeans that exerts preventive effects on bone loss in ovariectomized (OVX) animals. These effects have been correlated with increasing serum equol levels. In the present study, we investigated the effects of antibiotic intake on equol metabolism from daidzein, and the corresponding levels of bone loss in OVX mice.

**Methods:**

Eight-week-old female ddY mice (n = 42) were either ovariectomized (OVX) or subjected to a sham operation (sham). OVX mice were then divided into six dietary subgroups: control diet (control), 0.3 % kanamycin diet (KN), 0.1 % daidzein diet (Dz), 0.1 % daidzein and 0.0375 % kanamycin diet (Dz+KN3.75), 0.1 % daidzein and 0.075 % kanamycin diet (Dz+KN7.5), and 0.1 % daidzein and 0.3 % kanamycin diet (Dz+KN30). The mice were fed their respective diets for 4 weeks.

**Results:**

Uterine weight and femoral bone mineral density (BMD) were significantly lower in the OVX mice compared in the sham mice. No significant differences in uterine weight were observed among all OVX dietary subgroups. The Dz subgroup was found to exhibit higher plasma equol and *O*-desmethylangolensin (*O*-DMA) concentrations, as well as greater femoral BMD, compared to all other OVX subgroups. Furthermore, when compared to the Dz group, kanamycin intake decreased plasma equol and *O*-DMA concentrations, as well as femoral BMD in the OVX mice.

**Conclusions:**

These results suggest that kanamycin intake inhibited the conversion of daidzein to equol and *O*-DMA, blocking the preventive effects of daidzein on bone loss in OVX mice. Therefore, the bone-protective effects of daidzein intake may be predominantly associated with increased plasma concentrations of either equol or *O*-DMA.

## Background

Soybean isoflavones have molecular structures that are similar to estrogen, and exhibit a weak affinity for estrogen receptors [1]. Previous studies have demonstrated that soybean isoflavones prevent bone loss in ovariectomized (OVX) animals [2–6]. However, some intervention trials found positive effects of soy and soy isoflavones specifically on bone mineral density (BMD) and/or biomarkers of bone metabolism in pre- and postmenopausal women [7–9], whereas others have reported no significant effects or effects without clinical relevance [10, 11]. Because all subjects received minerals and vitamins such as calcium and vitamin D in clinical trials of Caucasian women, the effects of isoflavones on the bone remain unclear. This may explain the controversial results of various studies. Additionally, isoflavone metabolites can be produced, particularly equol from daidzein.

Daidzein is one of the predominant soybean isoflavones, and it is metabolized by gut microflora in the gastrointestinal tract to form equol or *O*-desmethylangolensin (*O*-DMA). Of these two metabolites, equol exhibits stronger estrogenic activity than daidzein [12]. We previously reported that soybean isoflavone intake increased plasma equol concentration, and inhibited bone loss in OVX mice, whereas fructooligosaccharides were found to stimulate the gut microflora leading to increased genistein, daidzein, and equol bioavailability [13, 14]. Furthermore, we reported that equol directly inhibited bone loss in OVX mice [15]. In a human study, we performed a 1-year double-blind randomized trial to compare the effects of isoflavone (75 mg of isoflavone conjugates/day) with those of placebo on BMD, fat mass, and serum isoflavone concentrations in early postmenopausal Japanese women who were classified based on their equol-producer phenotype [16]. Significant differences were observed between the equol producers and nonproducers in the isoflavone group with regard to the annualized changes in BMD and fat mass [16]. Therefore, it has been suggested that equol derived from daidzein may have a bone-protective effect.

Recent studies have suggested that the clinical effectiveness of isoflavones may depend on the ability of an individual to produce equol [12, 16–18]. Although only 30–50 % of the human population can produce equol [18–20], common laboratory animals consistently produce high levels of equol. Therefore, the daidzein-induced bone-protective effect cannot be evaluated in the presence of this innate equol production. Previous investigators have reported that kanamycin antibiotic treatment resulted in a marked reduction in plasma equol concentration in cynomolgus monkeys [21]. In the present study, we investigated the effects of kanamycin antibiotic intake on daidzein-derived equol production and bone loss in OVX mice.

## Methods

### Experimental design

The Animal Study Committee of the National Institute of Health and Nutrition approved this study. All procedures were undertaken in accordance with the Committee of the National Institute of Health and Nutrition Guidelines for the Care and Use of Laboratory Animals. Eight-week-old female ddY mice (n = 42) were purchased from Japan SLC, Inc. (Shizuoka, Japan) and individually cage-housed in a room maintained at 23 °C with a 12-h light/dark cycle. Mice received a sham operation (sham) or were OVX. Sham mice were fed a control diet based on the AIN-93G formulation described previously [22], but containing corn oil rather than soybean oil. OVX mice were divided into six dietary subgroups: control diet (control), 0.3 % kanamycin diet (KN), 0.1 % daidzein diet (Dz), 0.1 % daidzein and 0.0375 % kanamycin diet (Dz+KN3.75), 0.1 % daidzein and 0.075 % kanamycin diet (Dz+KN7.5), and 0.1 % daidzein and 0.3 % kanamycin diet (Dz+KN30). We chose kanamycin doses in the respective diets based on an experiment of cynomolgus monkeys [21]. The mice were fed their respective diets and allowed deionized water ad libitum throughout the study period. Animals were weighed by electronic balance at 10:00 in every week. After 4 weeks, the mice were euthanized by examination under anesthetized with pentobarbital sodium (40 mg/kg body weight) and sacrificed. Blood, uterine tissue, and femoral bone samples were collected for analysis.

### Radiographic analysis of the femur

Radiographic analysis of the right femur was performed using a soft X-ray system. The left femur was removed and stored in 70 % ethanol at 4 °C and dried at 60 °C overnight before analysis. Femoral bone mineral content (BMC) and BMD were measured by dual-energy X-ray absorptiometry (DXA) using the DCS-600EX-R system (Hitachi Aloka Medical, Ltd., Tokyo, Japan). BMD was calculated using BMC of the measured area. Intra-assay and Inter-assay coefficients of variation were less than 1.0 and 4.8 %, respectively. The detection limit of BMD was 15 mg/cm^2^. The mineralization profiles of the femur were stored from the monitor images, and the BMC and BMD values were obtained for the femur.

### Time-resolved fluoroimmunoassay (TR-FIA) for plasma daidzein, equol, and *O*-desmethylangolensin (*O*-DMA)

A TR-FIA was utilized to determine plasma levels of daidzein, equol, and *O*-DMA as described by Wang et al. [23], Brouwers et al. [24], and L’homme et al. [25], respectively. This volume corresponded to 20 μL of the original plasma sample. Samples showing a value outside the range of the standard curve were diluted with assay buffer. Another 20 μL of the solution was used for liquid scintillation counting to determine recovery. Based on these results, the final values were corrected for losses during hydrolysis and extraction. Before the assay, microstrips coated with goat anti-rabbit immunoglobulin G were prewashed using 1296–026 DELFIA platewash (Wallac, Oy Turku, Finland). A volume of 20 μL of the standard or hydrolysed and extracted plasma samples was pipetted onto the microstrips, then 100 μL of antiserum in 50 mM Tris–HCl buffer containing 5 g/L BSA (pH 7.8) for daidzein, equol, or *O*-DMA and 100 μL of europium-labelled daidzein, equol, or *O*-DMA was added per well. The strips were placed on a 1296–003 DELFIA shaker (Wallac) and shaken slowly at room temperature for 90 min, and then washed in the DELFIA platewasher (Wallac) using the no. 29-T3 program. A volume of 200 μL of DELFIA enhancement solution 1244–105 (PerkinElmer, Weltham, MA, USA) was added to each well, and the strips were shaken slowly for an additional 5 min. Fluorescence was read using the DELFIA Victor 1420 multi-label counter (Wallac) and the accompanying software (version 1.0) for data analysis. The final concentrations (Concs) were calculated using the following formula:

$$Conc\; = \;Conc\;(read)\; \times \;(1/re\text{cov} ery\;factor)\; \times \;dilution\;factor\;(nmol/l)$$

The average percent coefficient of variation (% CV) values for the daidzein, equol, and *O*-DMA assays are 4.0, 5.5, and 5.6 %, respectively [23–25].

### Statistical analysis

Data were expressed as the mean ± SEM. After performing a one-way analysis of variance, the statistical significance of any differences was determined using Fisher’s protected least significant difference test (StatView 4.0, Abacus Concepts, Piscaraway, NJ, USA). P values less than 0.05 were considered significant. Bars not sharing letters denote significant differences in figures.

## Results

### Body and uterine weight

**Fig. 1:**
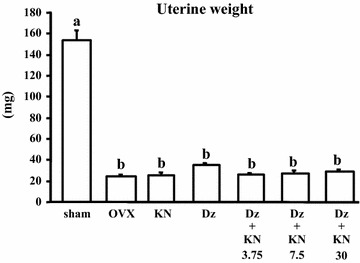
Uterine weight. The data are presented as the mean ± SEM for each group of 6 mice. *a, b Bars* not a sharing letters denote significant differences (P < 0.05)

Initial body weight did not differ among the groups. The final body weight was significantly higher (P < 0.05) in the OVX control group than in the sham group and other five OVX subgroups, but there were no significant differences among the sham and other five groups (data not shown). Uterine weight was significantly decreased by ovariectomy (P < 0.05), indicating that the mice were estrogen-deficient. Additionally, daidzein with or without kanamycin intake did not affect uterine weight (Fig. 1).

### BMC and BMD of the femur

**Fig. 2:**
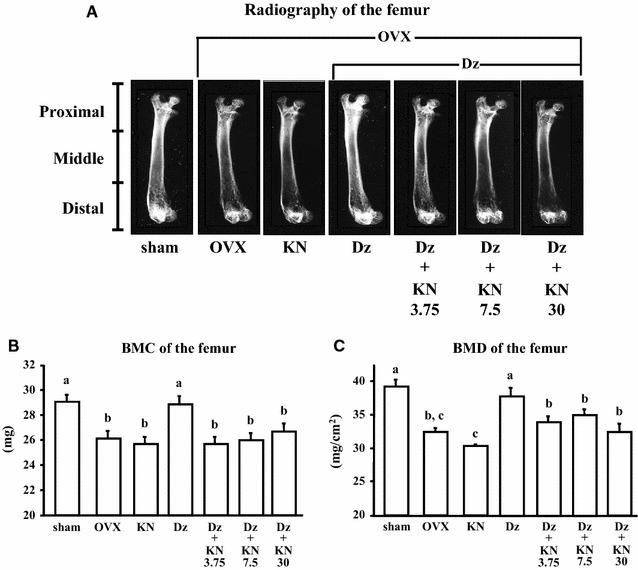
Radiography (**A**), BMC (**B**), and BMD (**C**) of the femur. The data are presented as the mean ± SEM for each group of 6 mice. *a–c Bars* not sharing letters denote significant differences (P < 0.05)

As shown by the radiography results, daidzein inhibited bone loss in the femur in OVX mice, while simultaneous intake of kanamycin did not rescue this bone loss (Fig. 2A). Femoral BMC was significantly lower in the OVX group than in the sham group (Fig. 2B). BMC of the femur in the OVX Dz subgroup was significantly higher than in the other OVX subgroups (P < 0.05), and there were no significant differences among the control, KN, Dz+KN3.75, Dz+KN7.5, and Dz+KN30 subgroups. In mice fed the daidzein-supplemented diets, femoral BMC was significantly lower in the kanamycin-treated groups (KN, Dz+KN3.75, Dz+KN7.5, and Dz+KN30) than in the Dz group (P < 0.05) (Fig. 2B). A similar tendency was observed for BMD (Fig. 2C).

### Plasma daidzein, equol, and *O*-DMA concentrations

**Fig. 3:**
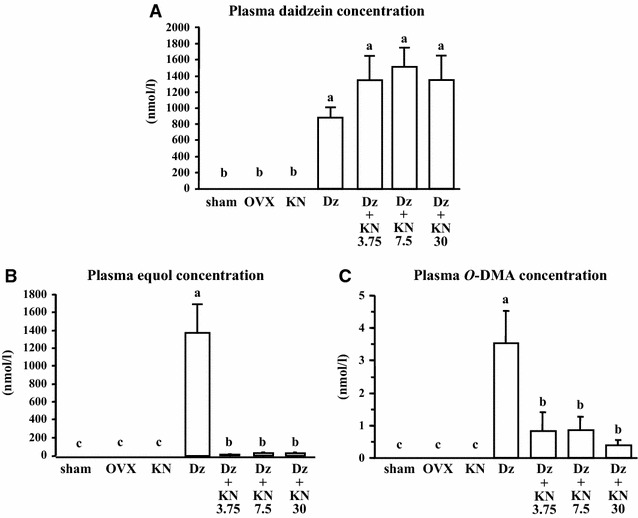
Plasma daidzein (**A**), equol (**B**), and *O*-DMA (**C**) concentrations. The data are presented as the mean ± SEM for each group of 6 mice. *a–c Bars* not sharing letters denote significant differences (P < 0.05)

Daidzein intake increased plasma daidzein, equol, and *O*-DMA concentrations in OVX mice (Fig. 3A–C). In the mice receiving daidzein-supplemented diets, kanamycin intake did not alter plasma daidzein concentration, but plasma equol and *O*-DMA concentrations decreased.

## Discussion

A previous study showed that daidzein prevented BMD decreases in the femur and the lumbar vertebrae of OVX rats without evidence of uterine hypertrophy [26]. In the present study, uterine weight as well as femoral BMC and BMD were significantly lower in the OVX group than in the sham group. Furthermore, daidzein intake inhibited BMC and BMD decreases in the femur, but had no significant effect on uterine weight in OVX mice. Isoflavones are candidate chemicals as selective estrogen receptor modulators (SERMs). SERMs are estrogen receptor ligands that act as estrogen on the bone, while blocking estrogen action in reproductive organs [27]. Although high isoflavone doses have been shown to induce uterine hypertrophy in OVX mice [3], the results of the present study suggest that appropriate daidzein intake improved BMC and BMD without uterine hypertrophy. The femoral radiographic images obtained using a soft X-ray system corroborated these findings (Fig. 2A).

Microflora in the gastrointestinal tract metabolize daidzein to produce equol or *O*-DMA [12, 19, 28]. Mice have an innate ability to produce equol [12], and therefore equol production was inhibited in some of the mice subgroups in this study by kanamycin antibiotic treatment. Kanamycin treatment has been shown to cause a marked reduction in plasma equol concentrations in cynomolgus monkeys [21]. Furthermore, Bowey et al. reported that equol and *O*-DMA were not detected in urine from germ-free rats [28]. In the present study, kanamycin treatment did not change the plasma daidzein concentration, but it did decrease plasma concentrations of equol and *O*-DMA in mice fed daidzein-supplemented diets. These results suggest that kanamycin intake interferes with the conversion of daidzein to equol and *O*-DMA in the gastrointestinal tract without alterating daidzein absorption.

In the present study, daidzein intake increased plasma concentrations of daidzein, equol, and *O*-DMA in OVX mice. In addition, femoral BMC and BMD were significantly higher in the Dz group than in the control group. We previously showed that equol prevented a reduction in femoral bone loss in OVX mice [15]. Furthermore, *O*-DMA exhibits an inhibitory effect on in vitro osteoclast formation [29]. Therefore, it is not possible to assess the daidzein-induced bone-protective effect in the presence of metabolites such as equol and *O*-DMA. Because kanamycin reduces the population of equol-producing bacteria, the metabolism of equol and *O*-DMA from daidzein may be inhibited during enterohepatic recirculation. Furthermore, bone loss was also observed in the kanamycin-treated OVX subgroups (KN, Dz+KN3.75, Dz+KN7.5, and Dz+KN30) despite daidzein supplementation. These results suggest that daidzein intake combined with kanamycin treatment does not inhibit reductions in femoral BMC and BMD in OVX mice. Thus, the bone-protective effects of daidzein intake may be primarily affected by either the equol or *O*-DMA metabolite. Based on the results of our previous study [29], equol may exert a greater effect on bone than does *O*-DMA. Taken together with the present study, equol may be the most effective compound in bone among the three isoflavonoids.

## Conclusions

In the present study, we utilized an OVX model in mice to demonstrate the effects of kanamycin antibiotic intake on equol metabolized from daidzein, and the impact on bone loss. Kanamycin intake inhibited increases in plasma equol and *O*-DMA concentrations, and femoral BMC and BMD decreased in the kanamycin-treated OVX subgroups despite dietary supplementation with daidzein. These results suggest that either equol or *O*-DMA may is a key factor related to the bone-protective effects of daidzein.

## Abbreviations

BMC: bone mineral content
BMD: bone mineral density
*O*-DMA: *O*-desmethylangolensin
OVX: ovariectomized
SERM: selective estrogen receptor modulator
TR-FIA: time-resolved fluoroimmunoassay

## References

[1] Morito K, Hirose T, Kinjo J, Hirakawa T, Okawa M, Nohara T, Ogawa S, Inoue S, Muramatsu M, Masamune Y (2001). Interaction of phytoestrogens with estrogen receptors alpha and beta. Biol Pharm Bull.

[2] Ishimi Y, Miyaura C, Ohmura M, Onoe Y, Sato T, Uchiyama Y, Ito M, Wang X, Suda T, Ikegami S (1999). Selective effects of genistein, a soybean isoflavone, on B-lymphopoiesis and bone loss caused by estrogen deficiency. Endocrinology.

[3] Ishimi Y, Arai N, Wang XX, Wu J, Umegaki K, Miyaura C, Takeda A, Ikegami S (2000). Difference in effective dosage of genistein on bone and uterus in ovariectomized mice. Biochem Biophys Res Commun..

[4] Wu J, Wang X, Chiba H (2004). Combined intervention of soy isoflavone and moderate exercise prevents body fat elevation and bone loss in ovariectomized mice. Metabolism.

[5] Uchida R, Chiba H, Ishimi Y, Uehara M, Suzuki K, Kim H, Matsumoto A (2011). Combined effects of soy isoflavone and fish oil on ovariectomy-induced bone loss in mice. J Bone Miner Metab.

[6] Santos MA, Florencio-Silva R, Medeiros VP, Nader HB, Nonaka KO, Sasso GR, Simões MJ, Reginato RD (2014). Effects of different doses of soy isoflavones on bone tissue of ovariectomized rats. Climacteric..

[7] Atkinson C, Compston JE, Day NE, Dowsett M, Bingham SA (2004). The effects of phytoestrogen isoflavones on bone density in women: a double-blind, randomized, placebo-controlled trial. Am J Clin Nutr.

[8] LaCroix AZ, Levy L, Li SS, Qu P, Potter JD, Lampe JW (2006). Soy protein and bone mineral density in older men and women: a randomized trial. Maturitas..

[9] Wu J, Oka J, Tabata I, Higuchi M, Toda T, Fuku N, Ezaki J, Sugiyama F, Uchiyama S, Yamada K, Ishimi Y (2006). Effects of isoflavone and exercise on BMD and fat mass in postmenopausal Japanese women: a 1-year randomized placebo-controlled trial. J Bone Miner Res.

[10] Lydeking-Olsen E, Beck-Jensen JE, Setchell KD, Holm-Jensen T (2004). Soymilk or progesterone for prevention of bone loss: a 2 year randomized, placebo-controlled trial. Eur J Nutr..

[11] Brink E, Coxam V, Robins S, Wahala K, Cassidy A, Branca F, PHYTOS Investigators (2008). Long-term consumption of isoflavone-enriched foods does not affect bone mineral density, bone metabolism, or hormonal status in early postmenopausal women: a randomized, double-blind, placebo controlled study. Am J Clin Nutr..

[12] Atkinson C, Frankenfeld CL, Lampe JW (2005). Gut bacterial metabolism of the soy isoflavone daidzein: exploring the relevance to human health. Exp Biol Med..

[13] Uehara M, Ohta A, Sakai K, Suzuki K, Watanabe S, Adlercreutz H (2001). Dietary fructooligosaccharides modify intestinal bioavailability of a single dose of genistein and daidzein and affect their urinary excretion and kinetics in blood of rats. J Nutr.

[14] Ohta A, Uehara M, Sakai K, Takasaki M, Adlercreutz H, Morohashi T, Ishimi Y (2002). A combination of dietary fructooligosaccharides and isoflavone conjugates increases femoral bone mineral density and equol production in ovariectomized mice. J Nutr.

[15] Fujioka M, Uehara M, Wu J, Adlercreutz H, Suzuki K, Kanazawa K, Takeda K, Yamada K, Ishimi Y (2002). Equol, a metabolite of daidzein, inhibits bone loss in ovariectomized mice. J Nutr.

[16] Wu J, Oka J, Ezaki J, Ohtomo T, Ueno T, Uchiyama S, Toda T, Uehara M, Ishimi Y (2007). Possible role of equol status in the effects of isoflavone on bone and fat mass in postmenopausal Japanese women: a double-blind, randomized, controlled trial. Menopause..

[17] Setchell KD, Brown NM, Lydeking-Olsen E (2004). The clinical importance of the metabolite equol-a clue to the effectiveness of soy and its isoflavones. J Nutr..

[18] Rowland IR, Wiseman H, Sanders TA, Adlercreutz H, Bowey EA (2000). Interindividual variation in metabolism of soy isoflavones and lignans: influence of habitual diet on equol production by the gut microflora. Nutr Cancer.

[19] Frankenfeld CL, Atkinson C, Thomas WK, Goode EL, Gonzalez A, Jokela T, Wähälä K, Schwartz SM, Li SS, Lampe JW (2004). Familial correlations, segregation analysis, and nongenetic correlates of soy isoflavone-metabolizing phenotypes. Exp Biol Med. (Maywood)..

[20] Setchell KD, Brown NM, Desai PB, Zimmer-Nechimias L, Wolfe B, Jakate AS, Creutzinger V, Heubi JE (2003). Bioavailability, disposition, and dose-response effects of soy isoflavones when consumed by healthy women at physiologically typical dietary intakes. J Nutr.

[21] Blair RM, Appt SE, Franke AA, Clarkson TB (2003). Treatment with antibiotics reduces plasma equol concentration in cynomolgus monkeys (*Macaca fascicularis*). J Nutr.

[22] Reeves PG, Nielsen FH, Fahey GC (1993). AIN-93 purified diets for laboratory rodents: final report of the American Institute of Nutrition *ad hoc* writing committee on the reformulation of the AIN-76A rodent diet. J Nutr.

[23] Wang GJ, Lapcík O, Hampl R, Uehara M, Al-Maharik N, Stumpf K, Mikola H, Wähälä K, Adlercreutz H (2000). Time-resolved fluoroimmunoassay of plasma daidzein and genistein. Steroids.

[24] Brouwers E, L’homme R, Al-Maharik N, Lapcík O, Hampl R, Wähälä K, Mikola H, Adlercreutz H (2003). Time-resolved fluoroimmunoassay for equol in plasma and urine. J Steroid Biochem Mol Biol.

[25] Lhomme R, Brouwers E, AlMaharik N, Lapcı´k O, Hampl R, Mikola H, Wähälä K, Adlercreutz H (2002). Time-resolved fluoroimmunoassay of plasma and urine *O*-desmethylangolensin. J Steroid Biochem Mol Biol..

[26] Picherit C, Coxam V, Bennetau-Pelissero C, Kati-Coulibaly S, Davicco MJ, Lebecque P, Barlet JP (2000). Daidzein is more efficient than genistein in preventing ovariectomy-induced bone loss in rats. J Nutr.

[27] Gapstur S, Morrow M (2001). Selective estrogen receptor modulation and reduction in risk of breast cancer, osteoporosis, and coronary heart disease. J Natl Cancer Inst.

[28] Bowey E, Adlercreutz H, Rowland I (2003). Metabolism of isoflavones and lignans by the gut microflora: a study in germ-free and human flora associated rats. Food Chem Toxicol..

[29] Ohtomo T, Uehara M, Peñalvo JL, Adlercreutz H, Katsumata S, Suzuki K, Takeda K, Masuyama R, Ishimi Y (2008). Comparative activities of daidzein metabolites, equol and O-desmethylangolensin, on bone mineral density and lipid metabolism in ovariectomized mice and in osteoclast cell cultures. Eur J Nutr..

